# Nanotechnologies in Pancreatic Cancer Therapy

**DOI:** 10.3390/pharmaceutics9040039

**Published:** 2017-09-25

**Authors:** Ayesha Manzur, Adeolu Oluwasanmi, Darren Moss, Anthony Curtis, Clare Hoskins

**Affiliations:** School of Pharmacy, Institute of Science and Technology for Medicine, Keele University, Keele, Staffordshire ST5 6DB, UK; ayeshamanzur@hotmail.com (A.M.); oluwasanmiadeolu@gmail.com (A.O.); d.moss1@keele.ac.uk (D.M.); a.d.m.curtis@keele.ac.uk (A.C.)

**Keywords:** pancreatic cancer, nanomedicine, drug delivery, nanoparticle, theranostic

## Abstract

Pancreatic cancer has been classified as a cancer of unmet need. After diagnosis the patient prognosis is dismal with few surviving over 5 years. Treatment regimes are highly patient variable and often the patients are too sick to undergo surgical resection or chemotherapy. These chemotherapies are not effective often because patients are diagnosed at late stages and tumour metastasis has occurred. Nanotechnology can be used in order to formulate potent anticancer agents to improve their physicochemical properties such as poor aqueous solubility or prolong circulation times after administration resulting in improved efficacy. Studies have reported the use of nanotechnologies to improve the efficacy of gemcitabine (the current first line treatment) as well as investigating the potential of using other drug molecules which have previously shown promise but were unable to be utilised due to the inability to administer through appropriate routes—often related to solubility. Of the nanotechnologies reported, many can offer site specific targeting to the site of action as well as a plethora of other multifunctional properties such as image guidance and controlled release. This review focuses on the use of the major nanotechnologies both under pre-clinical development and those which have recently been approved for use in pancreatic cancer therapy.

## 1. Introduction

Pancreatic cancer (PC) is the 4th common cause of cancer related mortality in the western world and is predicted to become the 2nd leading cause by 2030 [1]. Despite significant developments in the treatment of other cancer types, the 5-year survival rate for PC (approximately 5%) remains the same. Pancreatic adenocarcinoma (PDAC) makes up >90% of PCs [2] and is the most demanding PC to treat [3]. PDAC is the only gastrointestinal (GI) malignancy with the lowest survival and with the lowest quality prognosis [1]. PC is usually diagnosed at a later stage and is strongly resistant to chemo- and radiation therapy [4] due to lack of distinctive side effects. The mechanisms concealed in PC growth and progression are yet to be thoroughly clarified, obstructing the progress of treatment schemes for disease management. Major challenges of PC today are potential metastatic spread, high local recurrence and chemo-resistance induced by cancer cell stems [5]. Among the current crucial issues with PC today is that current CT and MRI imaging techniques are unable to precisely detect and visualise the cancer stage to obtain a measure of the extent of cancer burden, which would allow selecting a suitable treatment approach based on the cancer stage [6]. The death rate of patients suffering from PC is predicted to increase by a further 28% by 2026. 80% of the patients diagnosed with PC are predicted to die within 12 months of diagnosis while as little as 3% will live for 5 years [7].

**Figure 1:**
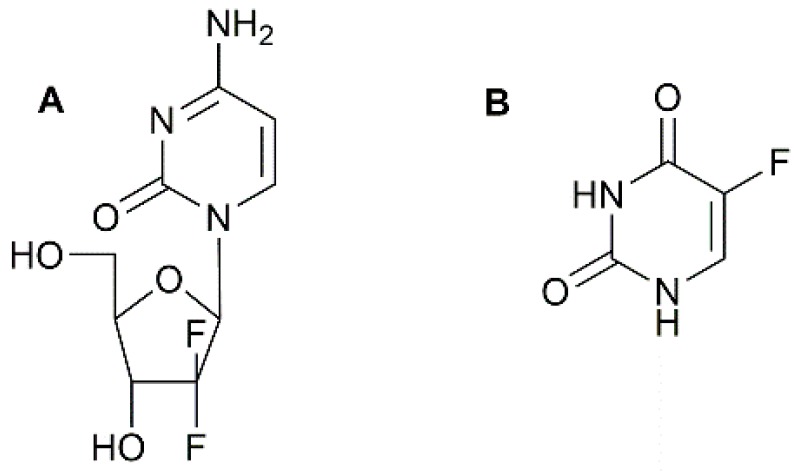
Chemical structure of (**A**) gemcitabine and (**B**) 5-fluorouracil.

For PC patients, the majority of the treatments currently accessible are palliative [8]. Gemcitabine (Figure 1A) is currently the first line treatment used for PC in the UK. Gemcitabine is a pro drug, which is a drug that is administered in an inactive form and phosphorylated into its clinically active form gemcitabine triphosphate within cells after cellular uptake [9,10]. This active form inhibits DNA elongation by replacing the nucleoside cytidine leading to cell death [11]. Gemcitabine therapy has been the primary regimen for metastatic or un-resectable locally advanced PC since 1997 [12]. Gemcitabine only takes effect in 23.8% of PC cases [13] believed to be the result of the dense stroma encasing the tumour and preventing drug penetration [14]. Without the prevalence of obvious biomarkers, 80–90% of PDAC occurrences undergo late diagnosis and hence surgical resection will no longer be successful. For the remaining (10–20%) for which it is possible, the patients have a high incidence of death due to recurrent or metastatic disease [15]. PDAC treatment has also failed due to chemo- and radiation resistance. A neoadjuvant therapy followed by surgical resection is a possible solution for tumour resection [16]. Precise chemotherapeutic agents such as gemcitabine can generate significant sensitisation in cancer cells to radiation making the gemcitabine-radiation combination the current best neo-adjuvant treatment [17,18,19]. However, few PDAC patients benefit from the gemcitabine chemo-sensitising benefits and there are no means of predicting which case will improve from combined treatment [16,20].

Examples of other drugs which have been used to treat PC include: 5-fluorouracil (5-Fu), cisplatin and capecitabine. These drugs all inhibit tumour growth by interacting with the processes involved in the synthesis of new proteins vital for cell proliferation. They also inhibit cell growth by also inhibiting DNA replication or causing enough stress to the cells leading to apoptosis [21]. Prior to the use of gemcitabine, 5-Fu (Figure 1B) was the first line drug for treating PC. 5-Fu is a thymidylate synthase inhibitor. This enzyme is responsible for the synthesis of thymidine which is a nucleoside required for DNA replication. After 5-Fu is administered, it is quickly metabolised to 5-fluorodeoxyuridine 5′-monophosphate (FduMP) which binds to thymidine synthase blocking its ability to methylate uracil. Uracil forms base pairs with adenine during DNA transcription. It is methylated to form thymine which is required for DNA replication. Without the ability to undergo DNA replication, the cell become starved of thymidine after multiple divisions [22]. 5-Fu can also be triphosphorylated and become incorporated into RNA in the place of uracil blocking DNA transcription which ultimately stops the growth of cancerous cells. Gemcitabine replaced 5-Fu because it displayed fewer and less serious side effects as well as a greater therapeutic effect [12]. Even with the increased survival rate with gemcitabine the prognosis for PC is still abysmal and urgent improvements and novel strategies in PC chemotherapeutics are needed.

Therefore, more effective strategies are required in order to fulfil the immediate need for more effective PC therapies. One rapidly emerging field in biomedicine is the development of nanotechnologies for drug delivery, image guidance and controlled release, which have shown huge potential for cancer therapy.

## 2. Nanotechnologies in Cancer

A vast array of research has been carried out using the principles of nanotechnology for therapeutic drug delivery. Each system has characteristics unique to itself, but in large, for cancer therapy the ultimate goal is to achieve enhanced drug absorption and permeability in addition to site specificity and control over drug release rate [23]. The use of nanotechnologies as drug chaperones has numerous advantages over traditional excipient technologies such as surfactants and emulsions. Nanoparticle systems can ‘escort’ drug molecules to the planned destination, avoiding the body’s natural barriers, hence preventing early drug degradation or metabolism. The size of NPs (1–100 nm) allows them to interact with biological molecules both within cells and extracellularly which is highly advantageous for cancer diagnosis [6,24] and therapy [6]. Nanocarriers are straightforward and inexpensive to synthesise and can easily be customised or tailored for the application. This may include incorporation of a specific functional group to aid in targeting, or features such as fluorescence or imaging capability in order to track their journey after administration through to the cellular level [25].

Nanoparticles are capable of being (a) passively targeted to tumour vasculature through enhanced permeability and retention effect (EPR) [26] or (b) actively aimed at specific targets after functionalising with site-specific ligands [27]. Previous research has shown that binding nanoparticles with cytotoxic agents can enhance tumour penetration and caused more successful treatments [28,29,30].

Embodying nanoparticle drug carriers with chemotherapeutic drugs has many advantages: Firstly, the circulation time of the drug increases significantly as drugs are loaded onto or into nanoparticles which can avoid them being quickly eradicated by liver and kidneys. Next, the size of the particles results in a passive targeting known as EPR. This phenomenon is caused when the rapidly forming tumour vasculature does not form properly which results in tiny pores into which nano-sized preparations can become accumulated. Due to the extremely poor lymphatic drainage in these areas the nanoparticles are unlikely to leak back out into the circulation and hence higher drug concentration is achieved at the tumour area. Finally, toxic side effects of adjuvants are prevented by avoiding the use of adjuvants completely as the often ‘difficult’ lipophilic drugs can easily be incorporated into or onto the nanoparticle system.

Another possibility for nanotechnology in site specific drug delivery is to exploit active targeting mechanisms. Here, specific ligands are attached onto the surface of the nanotechnologies such as peptides, aptamers or antibodies. Addition of these specific ligands will result in the preferential binding onto receptors on the targeted cells, hence they act as recognition moieties. This results in highly site-specific targeting, ensuring the nano-formulation reaches its targeted sites and evades other unwanted cells, resulting in more efficient treatment.

Numerous studies have been carried out using a wide range of nanotechnologies for treatment of numerous cancers including breast [31,32], ovarian [33,34], colorectal [35,36], prostate cancer [37,38] etc. The use of nanotechnologies for the enhanced pre-clinical treatment of PC has also been extensively reported. In this review, we will outline the varied architectures of nano-systems and describe how they have been used to improve pre-clinical outcomes when tested in vitro/in vivo against this aggressive disease. Those which have shown promise as drug carriers have been summarised in Table 1.

## 3. Physiology of Pancreatic Cancer

PC is caused by the formation of neoplasia within the intraepithelial regions of the pancreas [58,59,60]. The pancreas is located within the abdomen surrounded by several other organs, major blood vessels and tissue types. This allows PC to spread quickly to neighbouring areas [60]. Cancers can spread when a single cancer cell enters the bloodstream. They are quickly swept away and usually settle when they reach a capillary [61]. Afterwards they pass through the wall of the blood vessel and attach to other tissues, organs or bone forming secondary cancers. The process of cancer cells emigrating to new sites is highly unlikely because it is very stressful for the cells which nearly always perish before completing the journey. However, it only takes one out of the multitude of cancer cells to survive. PC cells have the advantage of being situated near many blood vessels, multiple organ and tissue targets as well as the lymphatic system. Therefore even with successful treatment PC still has the possibility of reoccurring from satellite sites [61,62].

Pancreatic adenocarcinoma is characterised by dense solid tumours. Within the tumour microenvironment, different cells exist including cancerous cells, non-cancerous fibroblast cells and extracellular matrix. These solid tumours hinder drug permeation, hence without access to the cancerous cells themselves, many drug treatments prove non-effective. It has been reported, in the case of these dense PC tumours, that nanotechnologies do not result in EPR accumulation [63,64,65]. Additionally, the ability of chemotherapies, even if they do penetrate through the dense tumour stroma to distinguish between the cancerous and the fibroblast cells is not possible and it is likely that the potent drugs will be directed into and kill the off-target cells, and hence the PC cells will continue to proliferate and tumour grow. Therefore, active targeting mechanisms are required in order to direct the drugs to the appropriate cells within this complex microenvironment.

Potential targets for active transport include adhesion molecules such as integrins [66], other adhesion molecules [67], antigens [68] and proteases [69] which are reportedly upregulated on the surface of PC cells. These molecules are expressed by stroma tissues and involved in the process of attachment of PC cells to the fibrous extracellular matrix. Hence, targeting these sites may disrupt the microenvironment while directing the drug to the specific cells which require treatment.

## 4. Nanotechnologies in Pancreatic Cancer

### 4.1. Polymer Drug Conjugates

**Figure 2:**
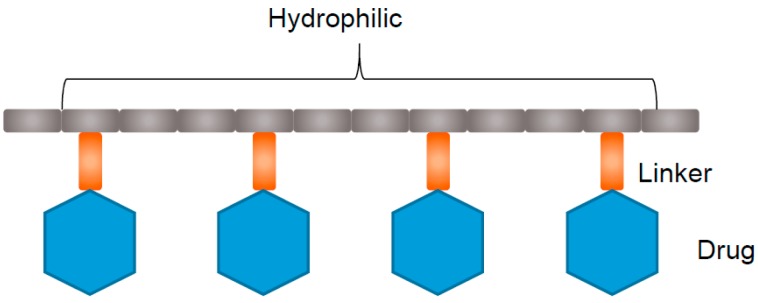
Schematic representation of a polymer drug conjugate.

Polymer drug conjugates are composed of drug molecules conjugated onto polymers (Figure 2), which can be applied in diagnosis and detection of cancer. They are characterised by a rationally designed covalent chemical bond between a hydrophilic polymeric carrier and bioactive molecule(s) [70]. Often anticancer therapeutics are highly lipophilic and hence cannot progress through clinical trial as administration becomes too difficult. However, by conjugating the drug onto the hydrophilic polymer backbone this results in the formation of core-shell aggregates, whereby the hydrophobic drug exists inside the lipophilic micellar core. Hence, this improves the aqueous solubility of the anticancer agent and alters drug pharmacokinetics at the whole organism and even subcellular level [39,71,72]. It also increases the chances to clearly enhance drug therapeutic effect.

A recent report showing a good example of this has been described by Wang et al. [39]. Here, a polymer-drug conjugate was developed between a poly(ethylene glycol) based polymer and 7-ethyl-10-hydroxycamptothecin (SN38): poly(ethylene glycol)-P(HEMASN38). SN38 is the active metabolite of irinotecan, which has shown promise as an anticancer agent for PC. SN38 cannot be administered in the free drug form to patients due to its poor solubility in both aqueous solutions and other pharmaceutically approved solvents [73]. Hence conjugation onto the poly(ethylene glycol) based polymer acts as a means to improve solubility and hence bioavailability. In addition to the SN38 conjugated onto the system, upon aggregation in aqueous media, vismodegib (GDC-0449) was loaded into the hydrophobic core, offering a dual therapy approach. GDC-0449 is a known hedgehog inhibitor for the treatment of basal carcinoma. The complex system demonstrated in vitro that the GDC-0449 reversed fibroblast-induced SN38 resistance and consequently a potent in vivo antitumour effect was achieved using the polymer-drug conjugate. Hence, the system may be applicable for future therapy of these solid dense tumours where drug penetration is an issue [39].

**Figure 3:**
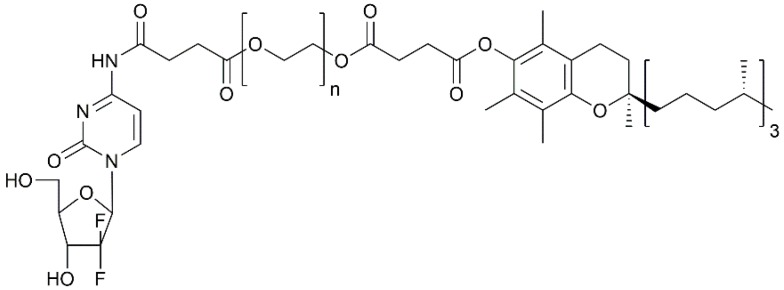
Chemical structure of tocopherol poly(ethylene glycol) succinate 1000-gemcitabine polymer drug conjugate.

Converse to most lipophilic cytotoxics, gemcitabine has a log P of 0.28 and does exhibit a reasonable degree of water solubility (0.8 mg·mL^−1^). As gemcitabine is the current first line therapy (with its associated flaws discussed earlier), the majority of the focus in polymer-drug conjugate formation for PC has been in the development of gemcitabine conjugated systems in order to increase drug efficacy [74,75,76,77,78]. Here the rationale for conjugation and micellar delivery is in order to protect the drug from premature degradation and promote elongation in circulation times, which may ultimately lead to increased therapeutic effect. Khare et al. reported the conjugation of gemcitabine onto D-alpha-tocopherol-poly(ethylene glycol) succinate (TPGS) (Figure 3) [40]. The study showed that after micellar formulation, the gemcitabine conjugated onto the TPGS exhibited a reduction in degradation (only 10% after 30 min) compared with free drug (89% after 30 min) due to cytidine deaminase. In vitro cytotoxicity studies showed a 1.5-fold reduction in cytotoxicity against human pancreatic adenocarcinoma (BxPC-3) cells. Gemcitabine usually enters the cellular lipid bilayer using nucleoside transporters. Hence, reduction in the availability of transporters or deficiency of their presence is one mechanism in which drug resistance may occur. Khare’s study showed that the polymer-drug conjugate was capable of enhancement in cytotoxicity when compared to free gemcitabine when inhibition of the nucleoside transporters had occurred. Hence, the authors concluded that the TPGS-gemcitabine polymer-drug conjugate held promise as a possible alternative strategy for gemcitabine delivery moving forward [40].

Recently, Joubert et al. fabricated a gemcitabine polymer-drug conjugate for prolonged cytotoxicity against PC [41]. The system developed was based on a methacrylate reversible addition-fragmentation polymerization (RAFT), which would control the release rate of gemcitabine upon hydrolysis of the ester and the amide link over long time periods. The study showed that release rate was significantly increased when the pH decreased from pH 7 (20% after 30 days) to pH 5 (50% after 30 days). The polymer-drug conjugate exhibited a higher IC_50_ value in Mia PaCa-2 cells compared with the free gemcitabine, however, the study was measured over 72 h and most likely the gemcitabine had not been released from the polymer structure after this short duration, as observed in the release studies. Hence, the cell viability was measured over 30 days. Interestingly, after 24 days the free gemcitabine had completely lost all cytotoxic effect on the cells with 100% viability being observed. However, in the cells treated with the polymer-drug conjugate, the cytotoxicity was maintained over the 30-day period. These findings show the use of nanotechnology in formulating sustained release systems which may prove more valuable compared to the current treatments due to their ability to maintain an effect over long time periods [41].

Mittal et al. developed a polymer-drug conjugate composed of gemcitabine conjugated onto a cationic poly(ethylene glycol)-*block*-poly(2-methyl-2-carboxyl-propylenecarbonate)-*graft*-dodecanol-graft-cationic ligand [42]. The system was used not only to deliver the anticancer agent but also to complex with a tumour suppressor miRNA-205 mimic. The system fabricated consisted of a gemcitabine >10% which was capable of 4/1 polymer/miRNA-405 mimic complexation rate. The system was tested in Mia PaCa-2 and CAPAN-1 cell lines in vitro and showed that the formulation effectively reversed chemo-resistance, invasion and migration of the cells compared to free gemcitabine. In vivo, a significant tumour growth inhibition was shown in Mia PaCa-2 xenografts with tumour weight after study end reducing from 0.53 g (free gemcitabine) to 0.14 g with the polymer-drug conjugate containing the miRNA-205 [42].

Another type of polymer, which fits into this class and had exhibited good potential as a cancer therapeutic, is polymer-enzyme conjugates. Provenzano et al. reported the use of a poly(ethylene glycol) linked with human recombinant PH20 hyaluronidase (PEGPH20) [79]. PH20 is an enzymatic agent which targets the hyaluronic acid in the desmoplastic stroma in PC tumours. Systemic administration of PEGPH20 in amurine PC xenograft resulted in a large decrease in tumour stroma as well as a decrease in interstitial tumour pressure with an increase in the lumen diameter of the blood vessels in the tumour [79]. PEGPH20 moved into Phase II clinical trial, however this was halted when concerns over the observation of thromboembolic events in some patients. To circumvent this heparin is now administered with the therapy. PEGPH20 is currently undergoing Phase III trial given in combination with gemcitabine-nab-paclitaxel in a biomarker selected patient population with HA-high levels [80].

### 4.2. Amphiphilic Polymers

Amphiphilic polymers contain both hydrophobic and hydrophilic moieties, which spontaneously aggregate into core-shell structures in aqueous environments [81]. This phenomenon is exploited in drug delivery for the solubilisation of lipophilic compounds inside the hydrophobic core of the aggregate formed. Amphiphilic polymers can exist in many different architectures dependant on their molecular formation. These include block copolymers, graft polymers and dendrimers. Of all the nanotechnologies described in this review, amphiphilic polymers represent the largest class of technology which has been investigated for PC therapy.

#### 4.2.1. Block Copolymers

**Figure 4:**
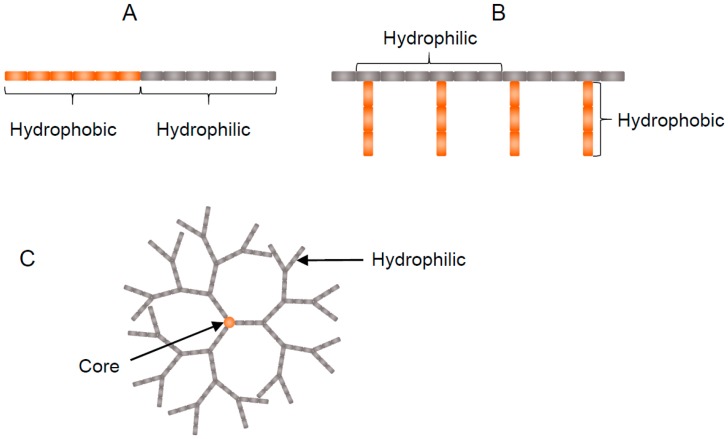
Schematic representation of amphiphilic polymer architectures: (**A**) Block copolymer; (**B**) Graft polymer and (**C**) Dendrimer.

Among the amphiphilic polymers, the most common type is block copolymers. Block copolymers are fabricated from the polymerization of two or more types of monomer units in a linear fashion (Figure 4A) [82]. Typically, one hydrophobic and one hydrophilic moiety becomes polymerised resulting in an amphiphilic diblock copolymer e.g., poly(ethylene oxide)-*block*-poly(lactic acid) (PEO-*b*-PLA) [83]. However, triblocks, tetrablocks, pentablocks etc., may also exist depending on the monomer ratio. In aqueous solutions, block copolymers form aggregates spontaneously to form polymeric micelles.

Characteristics of the micelles formed depend on the hydrophobic and hydrophilic monomer blocks. Ethylene glycol is the most commonly used hydrophilic monomer. In its polymeric form poly(ethylene glycol) is highly hydrated [84], an efficient steric protector [85], biocompatible [86] and it has also been shown to prolong circulation times in blood [87]. The most commonly used polymers for hydrophobic core formation are poly(esters) and poly(amino acids) [88,89]. The stability of polymeric micelles depends on the type and molecular weight of the hydrophobic block. Generally, the more hydrophobic and the higher the molecular weight, the greater the stability. In PC therapy, block copolymers have shown to solubilise a plethora of potent anticancer agents for disease treatment [43,44,45,90,91].

Kesharwani et al. reported the development of a styrene-maleic acid copolymer for the solubilisation and delivery of 3,4-difluorobenzylidene curcumin (CDF) [43]. CDF is a non-toxic analogue of curcimin, which in previous studies has shown to exhibit increased circulation times and specific accumulation in the pancreas [92,93,94]. Tailoring of the block copolymer was undertaken in order to achieve CDR loading of 5% to 15%. The polymer itself was found to release drug in a sustained manner over a period between 100–120 h dependant on pH. The novel formulation was tested on Mia PaCa-2 and AsPC-1 cell lines showing IC_50_ values relatively better than for the drug alone. The high level of cellular internalisation of the formulation meant that it produced a promising cytotoxic response, and further is being made to push this technology further into in vivo trials [43].

Recently, Veeren et al. reported the use of mixed micelles incorporating various anticancer agents (gemcitabine, doxorubicin, doxorubicin hydrochloride, 5-fluorouricil and paclitaxel) for PC therapy [45]. The mixed system was formed of poly(vinyl pyrrolidone-*b*-polycaprolactone) (PVP-*b*-PCL) and poly(vinyl pyrrolidone-*b*-poly(dioxanone-co-methyl dioxanone)) (PVP-*b*-P(DX-co-MeDX). As expected the more hydrophobic drugs encapsulated into the hydrophobic core of the micelles with greater efficiency than the less hydrophobic ones. Conversely, the more hydrophilic the drug molecule, the more rapidly release occurred into the aqueous surroundings. The toxicity of the nano-micelles was tested on BxPC-3 and Panc-1 cells: here, the data suggested that the empty carriers themselves were not toxic and hence any increase in toxic effect exhibited after formulation of the anticancer agents was solely down to greater internalisation. In vitro studies showed that the formulation of both gemcitabine and doxorubicin hydrochloride into one set of mixed micelles exhibited a greater extent of toxicity compared to the exposure of the mixed free drugs or the two drugs incorporated independently into micelles and exposed to the cell lines. These findings were independent of cell line or micelle type. Hence, more work is being carried out in order to determine the cause of the synergy and further potential of the system in vivo [45].

Other studies have shown the use of block copolymer micelles with specific functionalities. Ahn et al. reported the use of poly(ethylene glycol)-*b*-poly(glutamic acid) micelles for complexation of metal based oxaliplatin anticancer agent [44]. After loading the surface of the micelles they were conjugated with antibody fragments (Fab’). The study aimed to improve the efficacy of the Fab’ as a drug tumour targeting agent. After BxPC-3 cell exposure to the formulation, the data showed that the antibody conjugated-drug loaded micelles resulted in a 15-fold increase in cellular binding and more rapid internalisation compared to the non-antibody conjugated formulation. The formulation was tested in BxPC-3 xenografts, here a significant tumour inhibition was observed over 40 days which resulted in a significant improvement compared to both the free drug and the unconjugated counterpart [44]. These findings highlight how block copolymers can be used in order to promote long-term tumour retardation compared to existing therapies.

#### 4.2.2. Graft Polymers

Graft polymers or comb shaped polymers are branched polymers comprising of a hydrophilic homopolymer backbone with one or more hydrophobic pendant groups attached (Figure 4B) [95]. Like block copolymers, in aqueous environments these amphiphiles spontaneously aggregate into self-assemblies due to a reduction in Gibbs free energy. Commonly graft polymers are formed from cationic polymers with high molecular weights such as poly(ethylenimine) [96], poly(allylamine) [97] and chitosan [98] etc. Due to their relatively recent development, not many reports on the use of graft polymers for PC therapy have been reported.

**Figure 5:**
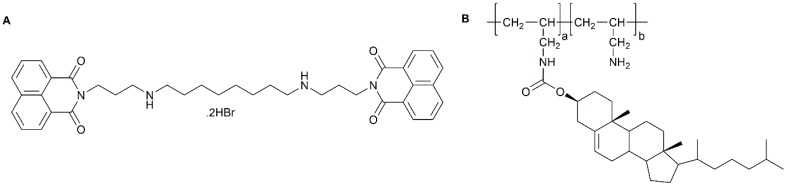
Chemical structure of (**A**) poly(allylamine)-cholesterol and (**B**) bisnaphthalimido propyldiaamino octane.

Hoskins et al. developed a graft amphiphilic poly(allylamine) derivative capable of solubilising hydrophobic drugs [46]. The polymer consisted of cholesteryl groups grafted in a 5% molar ratio onto the cationic polymer backbone forming (poly(allylamine)-*g*-cholesterol) (Figure 5A). The novel nano-carrier was used to solubilise a novel bisnaphthalimide based drug: Bisnaphthalimido propyldiaamino octane (BNIPDaoct) (Figure 5B). BNIPDaoct had shown promise in vitro against BxPC-3 cells, however, due to its inability to dissolve in aqueous formulations its progress was limited. The study showed that BNIPDaoct was capable of incorporation into the hydrophobic core of the micelle up to 0.3 mg·mL^−1^ at 1 mg·mL^−1^ polymer concentration and 1:1 polymer:drug loading ratio. The formulation was administered in vivo on a BxPC-3 xenograft in nude tumour bearing mice over a period of 4 weeks. Although the BNIPDaoct dose was eight-fold less than clinically used gemcitabine, the formulation was able to reduce tumour growth in xenograft mice with comparable results [46].

Bao et al. developed a chitosan grafted with poly(ethyleneimine) arms and candesartan conjugate (CPC) for targeted delivery to PC cells [99]. The candesartan (CD) was capable of specific binding onto angiotensin II type 1 receptors, which are over expressed in PC cells. The self-assemblies were complexed with w-p53 genes. When exposed to Panc-1 cells there was a preferential uptake into cells and a synergistic angiogenesis suppression was observed. The data showed that in Panc-1 xenografts the CPC/wt-p53 formulation significantly inhibited tumour associated angiogenesis. This harmonised system of halting tumour growth and decreasing tumour size may contribute to better success in the development of targeted tumour treatment [99].

#### 4.2.3. Dendrimers

Dendrimers are a category of repetitive, highly branched and thoroughly symmetrical polymers (Figure 4C). The number of repeating branch points as traced from the dendrimer centre to the periphery is called the generation. Dendrimer branches can act as selective gates to control the entry of small molecules [100]. Terminal groups on the dendrimer boundary can be adjusted to manage the solubility [101] and used as anchor points to enable functionalization with drugs, antibodies and polymers [102,103].

Amphiphilic dendrimers form unimolecular micelles when in aqueous environments. The mechanism of aggregation is dependent on the molecular makeup of the system but could involve hydrophobic or electrostatic interactions or hydrogen bonding [104]. The hydrophobic core of a dendrimer is capable of the encapsulation of hydrophobic drugs with controlled release profiles [105]. Entrapment can also be achieved within the multivalent branching network and/or via adsorption onto the outer shell [106]. When a drug is covalently attached to the outer shell of a dendrimer, it will exhibit a decreased release rate (if any—dependant on the binding mechanism) when compared to drug encapsulation by hydrophobic or electrostatic interactions [107]. Dendrimers with high drug conjugation have been shown to swiftly enter the cell and co-localise in the nucleus [107]. Of all the polymer nanotechnologies, dendrimers are by far the smallest, with average sizes ranging from 2–20 nm dependant on structure.

Often with nanotechnologies, increase in drug efficacy can be attributed to increased (a) rate of or (b) amount of drug internalisation into cells, which is likely to be influenced by uptake mechanism. It is widely reported that endocytosis is the main route of nanotechnology internalisation. However, this can be dependent on multiple factors including particle size, morphology, surface charge etc. As such, studies have been conducted into the transfer of third generation poly(amidoamine) (PAMAM) dendrimers labelled with Alexa Fluor 555 dye into Capan-1 cells visualised using confocal microscopy [108]. Poitz et al. reported that after microscopy analysis the data was input to a simple mass transfer model, which showed that the dendrimer was found to partition preferentially into the cells with a mass transfer coefficient of 0.054 µm/min. This finding was comparable with other studies where endocytosis was the uptake mechanism [108].

PC is one of the toughest cancers to treat due to its dense stroma hindering drug penetration. Any method to improvement the uptake of drug into tumour cells may offer beneficial outcomes. Kesharwani et al. as a follow-on study to the CDF loaded nanomicelles mentioned earlier in this review, have shown that PAMAM dendrimers with hyaluronic acid conjugation were capable of drug solubilisation and targeted delivery of CDF [47]. PC cells are known to overexpress CD44 (an integral membrane glycoprotein) on their surfaces [109,110]. As mentioned previously, CDF is a derivative of curcumin with 16-fold longer half-life. As such, formulation into a dendrimer would render the drug analogue soluble and would allow for clinical translation. Kesharwani reportedly achieved up to 17% entrapment efficiency of drug. Hyaluronic acid is capable of recognising and binding onto the CD44 glycoproteins and hence exploitation of active transport may result in more effective therapies. When the formulation was exposed to Mia PaCa-2 and AsPC-1 cell lines, a dose responsive toxicity was observed. In order to determine whether the hyaluronic acid was effective as a targeting agent, the CD44 in Mia PaCa-2 cells were blocked (saturated) by pre-treatment. The data showed that when the glycoproteins were blocked, a 1.71-fold increase in IC_50_ was observed compared to the untargeted formulation. This finding suggests that hyaluronic acid was indeed effective as an active targeting agent for PC treatments [47].

Other studies have highlighted the potential of Flt-1 as a target for site-specific delivery to PC tumour cells [48]. Flt-1 is a vascular endothelial growth factor (VEGF) receptor: these growth factors are secreted in order to induce capillary growth, thus providing the tumour with a vasculature blood supply. Hence, inhibition of this receptor would have a downstream effect and may result in more positive treatment regimes. Ozturk et al. reported the use of poly(ethylene glycol) cored PAMAM dendrimers with poly(ethylene glycol) surface modification. Additionally, anti-human Flt-1 was conjugated onto the dendrimer surface. The dendrimer system was used as an inclusion complex for incorporation of gemcitabine hydrochloride. The study showed that the gemcitabine was successfully incorporated into the structure with a loading efficiency of 5. The release profile showed a steady release over 20 h. When exposed to CFPAC-1 cells, the novel formulation experienced enhanced cytotoxicity compared to the free drug. Additionally, in xenograft studies increased accumulation was observed in tumour tissue. The authors concluded that this system may hold potential for those patients who do not respond well to conventional chemotherapies due to its highly targetable nature [48].

#### 4.2.4. Smart Polymers

Over the past two decades polymer science has gone from strength to strength. As scientists constantly strive to achieve more control over the site and or rate of delivery of therapeutics in order to increase efficacy and reduce patient side effects [111,112,113,114]. Stimuli responsive or ‘smart polymers’ have emerged as new front runners in the race for the ideal delivery system. Stimuli responsive polymers undergo active responses (conformational change) to environmental change or external signals. This makes them ideal as candidates for the active targeting of pharmaceuticals [114,115]. The reported stimuli may either be physical (temperature, ultrasound, light), chemical (pH, ionic strength) or biological (biomolecules) [114,116,117,118]. The major application reported for such technology is in the delivery of anticancer therapeutics where precision control and site specificity is the ultimate desire.

Temperature responsive polymeric micelles are the most extensively studied to date [119,120,121]. Cancerous tissues possess an increased metabolic rate compared to normal healthy tissues [122] and hence they exist at higher temperatures of 40–44 °C [122]. This highly localised temperature increase renders thermo-responsive polymers a promising choice of drug carrier. Once the nano-carrier has become internalised within the cell, the local temperature will increase which will result in polymeric micelle deformation and hence drug release will occur.

Recently, Emamzadeh et al. presented the development of thermo-responsive copolymers with a hydrophilic thermo-responsive block and a hydrophobic block [49]. The polymers formed exhibit a thermo-reversible phase transition above 37 °C triggered by an external temperature stimulus (such as ultrasound) resulting in micellar disruption and drug release. The polymers were composed of poly[(di(ethylene glycol) methyl ether methacrylate-*co*-poly(ethylene glycol) methyl ether methacrylate 300)-*b*-poly(2-ethylhexyl methacrylate)] [poly(diEGMAco-OEGMA300)-*b*-PEHMA]. The self-assemblies formed in aqueous environments were capable of incorporating amino-protected gemcitabine derivative, squalenoyl-Gemcitabine (sq-Gem) and PTX into their hydrophobic core. The self-assemblies developed exhibited conformation change at 40 °C. In vitro cytotoxicity assays carried out in Mia PaCa-2 cells, showed that combination therapy of the two cytotoxic drugs within the self-assemblies exhibited a synergistic effect with considerably decreased cancer cell viability. This study shows the great potential of thermo-responsive polymers in PC treatment [49].

pH responsive micelles can also be exploited as carriers in cancer treatment. The external pH of tumour tissue has been reported to be around pH 6.75 as compared to pH 7.4 for normal healthy tissue cells [115,116]. Additionally, the uptake mechanism of nanoparticles is largely reported to be predominantly through the endocytic pathways. Once inside an endosome, the pH drops from pH 7.4 to approximately pH 4–6 [123]. Hence, tailoring the pH at which polymer breakdown or deformation occurs can result in a highly site specific controllable release profile.

Li et al. developed an amphiphilic polymer from poly(styrene-alt-maleic anhydride) conjugated through 2,4-diaminobutyric acid linkers with additional folic acid surface modification for targeting of the folate receptor proteins up-regulated in PC cells [50]. The self-assemblies formed were capable of forming in neutral pH, under which condition drugs could be incorporated into their hydrophobic core. Upon reduction in pH, the polymer was unstable and collapsed resulting in drug release. In this study, curcumin was used as a drug mimicking molecule due to its high level of lipophilicity. Once exposed to Panc-1 cell lines, fluorescent microscopy confirmed that significant levels of curcumin had been internalised compared to those aggregates without folic acid tags. Interestingly, the self-assemblies appeared to exhibit rod-like association specific to the cells. The authors concluded that the system developed possessed excellent potential as a site-specific pH responsive delivery system for PC therapy [50].

Other studies have shown preferential drug release after micellar exposure to high frequency ultrasound. Rapaport et al. developed paclitaxel-loaded Perfluorocarbon (PFC) nanodroplets in combination with a PEO-co-PLA block copolymer, which they used with focussed ultrasound treatment for PC treatment [124]. The time period between drug administration and application of the magnetic resonance imaging guidance focussed ultrasound (MRgFUS) was proposed to have a remarkable function in positive PC therapy. This was because the formulation required time to collect at the tumour area to allow a suitable drug concentration to be present for release before MRgFUS application. By not considering the time factor this would have caused damage to healthy tissue [124].

In another study, Rapaport et al. also synthesised a novel-intravenous paclitaxel loaded nano-emulsion [51]. This nano-emulsion provided chemotherapy for PC via a conversion method system from nano-emulsion to microbubbles through application of ultrasonic energy (90 KHz–1 MHz). The tumour size decreased by the ultrasound activated system whereas the nano-emulsion not carrying the paclitaxel had no effect on the tumour size. This demonstrated that the outcome was due to the ultrasound-activated chemotherapeutic activity of the targeted paclitaxel and not solely on the mechanical or thermal effect of the ultrasound [51].

### 4.3. Albumin

Albumin is the most abundant plasma protein used in the body for the transport of nutrients; this is facilitated by its multiple binding sites and long circulatory half-life of approximately 19 days [125]. Albumin is well known as an effective drug carrier for lipophilic molecules [126] and has shown the greatest success in PC treatment to date. Nanoparticle-albumin bound paclitaxel (Abraxane^®^) was approved by the FDA in 2013 for treatment of metastatic PC. Abraxane^®^ has demonstrated anticancer action in preclinical studies as a single agent and cooperative activity jointly with gemcitabine in murine PC models [52,53]. When administered in combination with gemcitabine, Abraxane^®^ was found to enhance the intra-tumoural concentration of gemcitabine [52,53].

Another international, large-scale, Phase III study demonstrated that Abraxane^®^ in combination with gemcitabine is a significantly better therapy than gemcitabine on its own [127]. The combined therapy showed noticeable improvement in survival in PC patients at all time points. Presence of Abraxane^®^ improved the cumulative delivery of gemcitabine. Longer treatment period and higher cumulative dose in the Abraxane^®^/gemcitabine combination compared to gemcitabine alone demonstrated that this formulation can be administered efficiently [127].

Kim et al. have constructed an albumin nanoparticle system for co-delivering curcumin and paclitaxel (PTX), both of which possess significant anti-tumour activity for PC, through nanoparticle bound albumin PTX/curcumin [128]. The particles were formed using high-pressure homogenisation. The results of the study demonstrated that the formulation effectively released both drugs over a 24 h period. Cellular internalised into Mia Paca-2 cells resulted in a 71% lower IC_50_ after exposure to the combined treatment compared to particles only containing PTX. This technology showed the clinical potential of an enhanced anticancer pharmaceutical agent to synergistically halt pancreatic tumours and may lead to lower chemotherapy dosage in the future [128].

### 4.4. Inorganic Nanoparticles

Inorganic nanoparticles are arising as novel materials for biomedical applications due to their distinct magnetic, electrical, optical and electrochemical properties [129] and their ability to possess multifunctional properties [130]. The list of inorganic nanoparticles currently being fabricated with biomedical application and hence potential as future PC therapies is growing rapidly. In this review, we will cover the predominating particles showing potential in PC.

#### 4.4.1. Carbon Nanotubes

**Figure 6:**
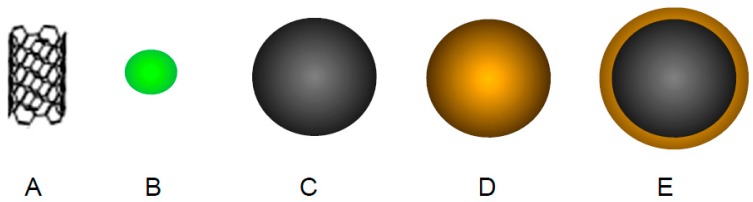
Schematic representation of inorganic nanoparticles: (**A**) Carbon nanotube; (**B**) Quantum dot; (**C**) Iron oxide; (**D**) gold and (**E**) iron oxide-gold.

Carbon nanotubes (CNTs) possess unique properties which can be harnessed for drug delivery (Figure 6A). These include a good degree of biocompatibility [131] as well as their exceptional function as drug conveyers with high target specificity and sensitivity [132]. CNTs have demonstrated to be efficient transporters to the specific target regions for a wide range of molecules including drugs, vaccines, proteins, small peptides, nucleic acids, vitamins and carbohydrates. The molecules are bound either by attachment onto the outer surface or incorporated into the inner cavity of the tubes. CNTs with surface functionalization have been shown to be more favourable in terms of biocompatibility and ability to internalise into cells [133,134].

Andreoli et al. reported the synthesis of a highly purified cationic CNT based on a single walled tube coated with poly(ethylenimine) [135]. The study aimed to determine whether the cationic CNTs were capable of entering the cells and assess their biocompatibility. In vitro studies in BxPC-3 cell lines showed that the CNTs coated with lower molecular weight poly(ethylenimine) resulted in higher biocompatibility; this was confirmed in vivo in pancreatic xenografts, whereby the high molecular weight PEI coated CNTs were found to cause local inflammation leading to necrosis in vivo. Shorter CNTs with lower molecular weight PEI coatings were developed which did prove biocompatible and showed colocation in both the cytoplasm and nucleus in PC cells [135]. This study shows the importance of physical parameters when developing inorganic nanoparticles for medical use, as they tend to carry a higher toxic burden than the synthetic polymers (though not in all cases).

CNTs have been reported in the literature as photo-thermal heat sources after exposure to near infrared irradiation [136]. Mocan et al. developed poly(ethylene glycol) coated multi-walled CNTs. The CNTs were internalised into Panc-1 cells and subjected to irradiation at 808 nm. This irradiation resulted in a heating effect which led to mitochondrial membrane depolarization. This membrane depolarization activated the flux of free radicals within the cell, leading to cellular oxidative stress and cell death. The authors concluded that the important findings may influence future combined strategies with chemotherapies in order to produce more effective PC treatments [136].

Sophisticated CNT systems have also been developed which offer multifunctional properties. Wang et al. recently reported the development of theranostic CNTs for image-guided phototherapy of PC specifically in metastatic lymph nodes [137]. In this study, the multi-walled CNTs were filled with MRI contrast agent gadolinium nanoparticles, which had been encapsulated within polydopamine. The final construct was surface coated with poly(ethylene glycol). The CNTs were internalised into BxPC-3 cells and irradiated with a 808 nm laser source, causing them to experience up to 32.4 °C in heating. Mapping was achieved using MRI, but also visually it was obvious to the naked eye when tissue had been stained black due to CNT accumulation. In vivo, the CNTs demonstrated lymphatic mapping ability and photo-thermal ablation of both primary tumour sites as well as metastatic lymph nodes [137]. Such an advanced technology could give hope to the millions of PC patients, where cancer spread has occurred which dramatically reduces survival chances.

#### 4.4.2. Quantum Dots

Quantum dots (QDs), are nano-sized semiconductor crystals, which possess unique optical and electronic properties (Figure 6B) [138]. In particular, they exhibit bright and intensive fluorescence, which is not easily quenched, unlike conventional fluorescent tags used in biomedicine [138]. QDs are highly tunable and can offer near infrared (>650 nm) emission which is advantageous for tissue imaging. Additionally, more than one QD can be combined in solution or tissue and each can be excited using a single light wavelength and detected simultaneously for multiple assays. Hence, with such potential a growing area of focus for QDs is for use as theranostics.

Yong et al. developed indium phosphate-zinc sulfide QDs for PC imaging [139]. The QDs were prepared through hot colloid synthesis and coated with mercaptosuccinic acid for aqueous dispersion. The surface of the QDs was functionalised using anti-Claudin4 in order to confer site-specific targeting. Claudin 4 is known to be overexpressed in both primary and metastatic pancreatic tumours [140]. The QDs were incubated with Mia PaCa-2 and Panc-1 cells and viability determined over 48 h. Up to 100 mg·mL^−1^ the QDs were not shown to cause any significant decrease in viability over the study duration. Cellular uptake was observed using confocal microscopy. Those cells treated with the QDs showed excellent uptake with bright signals compared to their unlabelled (no anti-Claudin4) counterparts [139]. The ease of synthesis and functionalisation and targetability of the QDs indicates their potential as a diagnostic tool for PC.

Diagnosis of PC is often hindered by the lack of apparent biomarkers or indeed the means to either detect or analyse these. As such Lee et al. have developed a QD approach for quantitative molecular profiling of PC biomarkers [141]. The QDs developed consisted of a CdS core with a (Cd,Zn)S shell. The surface of the QDs was functionalised with 1-myristoyl-2-hydroxy-sn-3-phosphocholine/1,2-dipalmitoyl-sn-glycero-3-phosphoethanol amine-*N*-(lauroyl)-poly(ethylene glycol) to confer aqueous stability. Antibodies were conjugated onto the surface in order to confer site specific targeting to the prostate stem cell antigen (PSCA), claudin-4 and mesothelin (MSLN) biomarkers selected for the study. The QDs were exposed to a panel of PC cell lines including Mia PaCa-2, Panc-1 and Capan-1. Upon exposure to cells the QDs exhibited highly selective targeting with low levels of non-specific binding. The saturation of biomarkers on the cell surface was confirmed and an absolute quantity of biomarker per mm^2^ could be deduced which was confirmed by flow cytometry [141]. These findings are important as it offers a new approach for diagnosis of PC using colour coded quantum dots.

Other studies have been reported for the use of QDs for UV enhanced cytotoxicity. Here, CdTe QDs were coated with mercaptopropionic acid and introduced into Panc-1 cells [142]. Once internalised the QDs were exposed to UV radiation and the extent of cytotoxicity evaluated. The data showed that metabolic activity of those cells containing QDs decreased upon UV irradiation in an exposure duration dependant manner, while those cells treated to QDs and no irradiation experienced only slight variation in metabolic activity. Further investigations showed that cells with QD plus UV irradiation produced significantly increased levels of reactive oxygen species, which is known to be detrimental to cell survival. Hence, this approach could be a simple and cost effective strategy in PC eradication in vivo [142].

#### 4.4.3. Iron Oxide Nanoparticles

Iron oxide nanoparticles (IONP’s) have applications in many medically related fields such as cancer treatment, cancer detection, drug transportation, cell labelling and magnetic cell sorting (Figure 6C) [143]. IONPs can exist in multiple shapes such as spherical, hexagonal, cubic and also rods. IONP’s smaller than 20 nm are superparamagnetic which means that they gain magnetism under a magnetic field. Unlike ferromagnetic materials, they do not retain their magnetism after the magnetic field is removed [144,145]. Superparamagnetism only occurs with nanoparticles because their size is small enough to occupy a single magnetic domain. This magnetic ability allows IONPs to be useful as MRI contrast agents and can aid in cancer imaging and diagnosis. Superparamagnetic IONPs can also attach drugs onto their surface as well as being magnetically guided to their target. Once the field is removed the magnetism is lost and the IONPs will re-disperse in the surrounding tissue, thus preventing aggregation and the likelihood of macrophage clearance [146].

Superparamagnetic IONPs have been developed by Arachchige et al. to enable MRI tracking and to act as a drug carrier for doxorubicin [54]. Dextran coated IONPs were labelled with red fluorescing doxorubicin as well as a green fluorescing fluorescein tag in order to track the in vitro fate of the particles inside PC cells. The particles were incubated with Mia PaCa-2 cells. The results showed that even in the absence of a targeting ligand, cellular internalization of doxorubicin had occurred with a 20-fold increase compared with free drug. Additionally, the data showed that once inside the cell doxorubicin was cleaved from the surface of the IONP allowing it to become localised within the nucleus [54].

Recently, Trabulo et al. have developed an IONP with anticancer agent gemcitabine conjugated onto its surface [55]. Additionally, in order to confer targeting ability anti-CD47, a monocloncal antibody which inhibits the CD47 receptor (found on PC stem cells and not on normal pancreatic cells), was also surface conjugated. The formulation was incubated with Panc-1, Panc 215 and Panc 354 cell lines. The data showed that, although there was no increase in overall cytotoxic effect using the combined therapy compared to the free drug, the cellular uptake was improved and that both the gemcitabine and CD47 retained their biological functions [55]. In 2D cell culture it is difficult to predict in vivo fate and therefore this strategy may result in more site-specific delivery to patients and hence less systemic drug circulation leading to unwanted side effects.

Magnetic IONPs will generate heat when subjected to strong magnetic fields. Magnetic crystal suspensions of IONPs store the energy of alternating magnetic fields and release this energy as heat causing hyperthermic stress in cancer cells [147]. Rochani et al. developed a sophisticated platform based on IONP hyperthermia treatment of PC in conjunction with heat shock protein inhibition [148]. The IONPs used in this study, were coated with poly(lactic-*co*-glycolic acid). The surface was further modified with 17-*N*-allylamino-17-dimethoxygeldanamycin (17AAG). 17AAG is a heat shock protein inhibitor. Heat shock proteins are produced when cells are exposed to thermal increase, chemotherapy or other stressful events. The production of these proteins is a coping mechanism of the cell in order to avoid undue levels of stress and protect itself. The study showed that the coating concentration of polymer effected not only the physicochemical properties, such a dispersion stability and particle size, but also the magnetic ability of the particles which is required for hyperthermia. The findings suggested that the lower polymer concentration gave more favourable magnetic characteristics, perhaps due to the higher concentration forming a thicker layer surrounding the iron oxide core. The dispersions were incubated with Mia PaCa-2 cells and exhibited a time and dose dependant toxicity profile. After cellular internalisation the particles were subjected to an alternating magnetic field, resulting in localised heating of the nanoparticles and a reduction in cell viability of 75% compared to the control cells [148]. This technology shows the potential as an alternative strategy to chemotherapy for future clinical use.

Block copolymers based on poly(lactide-*co*-glycolide)-*b*-poly(ethylene glycol) incorporating iron oxide nanoparticles with a matrix metalloproteinase-9 (MMP-9) cleavable linker have been developed as an enzymatically activated platform [149]. MMP-9 is a protease which is upregulated in malignant tumours. Protease cleavage of the Magh@PNPs-PEG-RegaCP-PEG construct formed resulted in activation of the core components through removal of a poly(ethylene glycol) outer coating and internalisation of smaller nanoparticles with their payload into the cancer cells. This was observed using transmission electron microscopy from solution and light microscopy after internalisation into 9801 L pancreatic carcinoma cells. In vivo xenograft studies showed that the nanoparticle accumulated in a tumour specific- and MMP-9 dependant manner. Grünwald et al. concluded that this system holds good potential as a future therapeutic strategy for preferential targeting within PC tissue [149].

Another system developed exploiting similar mechanisms, is the cerium doped Fe_3_O_4_ with chitosan coating based nanoparticles: CAN-Mag@PNPs-PEG-REGAcp-PPEG/tPApep1_lac_ [150]. These particles were developed to target the galectins Gal-1, Gal-3 and Gal-4. These galectins are known to be overexpressed on the surface of PCs. The tPA-derived peptide incorporated into the structure was used as a binding site for the nanoparticles while a proteolytically cleavable poly(ethylene glycol) shell was used in order to prolong circulation and provide stealth properties before protease activation from the monocyte phagocyctic system. The nanoparticles were tested in vivo in Panc-1 xenografts in mice. The nanoparticles were well tolerated by the mice suggesting little associated toxicity. The study demonstrated that the nano-construct was capable of higher levels of accumulation in the subcutaneous tumours compared to the same nanoparticles without the outer shell. Additionally, magnetic resonance imaging showed greater tumour uptake in the orthotropic model compared with the subcutaneous tumour models, possibly due to the extent of vasculaturisation. The authors concluded that these nano-carriers held potential as protease activated drug carriers and further work is underway in order to fully exploit these systems for PC therapy [150].

#### 4.4.4. Gold Nanoparticles

Gold nanoparticles (Figure 6D) have shown promise as useful carriers for targeted drug delivery. Like IONP’s, gold nanoparticles also come in a variety of shapes, including spheres, rods and tetrapods. Gold as an element is generally regarded as non-toxic. It is a noble metal that has filled d orbitals. This makes gold resistant to oxidation, corrosion, and acidic conditions that can be found within the body. Gold can be strongly covalently bonded to thiol groups, an organic functional group that can be found on some drug compounds, or attached via the use of linkers [151]. Gold’s biocompatibility, ability to carry drug formulations, ease of synthesis and surface plasmon resonance properties make it a powerful tool in the treatment of PC. One characteristic of gold is its ability to absorb near infrared (NIR) radiation and generate heat due to surface plasmon resonance (SPR). SPR is the term used to describe the oscillation of free electrons when light is shone on a metal surface. These resonating free electrons produce an electromagnetic wave (light) that travels away from the bulk phase of the solid [152,153]. Generating heat using NIR radiation via SPR is ideal considering the high transparency of tissue at that wavelength. A laser emitting NIR radiation can be used to target a specific area where gold nanoparticles are situated, such as within a cluster of neoplasia in the pancreas. Heat generated from the photo-thermal effect will raise the temperature of the cancer cells thus killing them due to hyperthermia. Because the area of effect of heating using the laser is small, this also prevents damage being caused to nearby healthy tissues [154].

Mocan et al. showed that gold nanoparticles which undergo laser irradiation at 808 nm were capable of interfering with the mitochondrial electron transfer chain in PC cells [155]. This interference occurred through interaction of the activated electrons on the gold nanoparticle surface after laser irradiation with the electrons inside the mitochondrial membrane in PANC-1 cells. This phenomenon results mitochondrial membrane depolarization leading to lysis. The study also showed that internalisation and irradiation of gold nanoparticles led to the inactivation of the Bcl-2 anti-apoptotic proteins resulting in a decrease in MTP and ultimately necrosis. The authors concluded that this data may represent a major step forward in the development of mitochondria-targeted PC treatments using laser-activated gold nanoparticles [155]. Saha et al. reported the use of gold nanoparticles for reprogramming the tumour microenvironment and inhibiting tumour growth in PC models [156]. The study showed that the gold nanoparticles were capable of disrupting the communication between both Panc-1, AsPC-1 cell lines as well as fibroblast cells associated with PC (CAF19 and iTAF). This communication breakdown was a result of alteration of the cell secretome. In the CAF19 and iTAF, the gold nanoparticles were capable of reducing matrix deposition and hence inhibit tumour growth in vivo in orthotropic PC models [156].

#### 4.4.5. Hybrid Iron Oxide-Gold Nanoparticles

Hybrid nanoparticles are composed of an iron oxide core surrounded by a gold shell (Figure 6E). The use of hybrid nanoparticles in nanomedicine has been reported over the past 5–10 years. The nanoparticles themselves possess physical characteristics of both the iron oxide core (magnetism) and the gold shell (biocompatibility and ability to act as a nano-heater). Hence, these multifunctional platforms have proven useful in PC treatment.

Guo et al. reported the use of hybrid nanoparticles (GoldMag) as thermal ablation sites in for PC therapy [157]. The GoldMag itself did display a degree of cytotoxicity with 47% cell viability observed after 24 h incubation. The particles were internalised into Panc-1 cells and irradiated at 808 nm for 5 min. Thermal rise was observed up to 79.51 °C at 50 μg·mL^−1^. Photo-thermal ablation at the same concentration resulted in reduction of the cell viability with only 2.3% live cells remaining. Hence, the cytotoxic properties of the hybrid nanoparticles coupled with heat generation produced a significant decrease in PC cell proliferation [157].

Another study that evaluated the potential of poly(ethylene glycol) coated hybrid iron nanoparticles as heat triggers for PC therapy looked at both the in vitro and in vivo outcome of administered hybrid nanoparticles and production of heat shock proteins (HSP-27 and HSP-70) which are produced under heat-related stressful conditions in cells [158]. These proteins are classed as molecular chaperones that assist various protein folding processes such as the folding of newly synthesised proteins and the refolding of misfolded proteins [159]. In this study, the hybrid nanoparticles were internalised into BxPC-3 cells and irradiated at 1064 nm for 60 s (or multiples thereof). The data showed that the particles were capable of thermal increase of up to 9 °C with heat dissipation over a total area of 346 mm^2^. Significant elevation in levels of the HSPs after laser irradiation (50 µg·mL^−1^ HNP) indicate that the cells are stressed by the increase in the heat generated by surface plasmon resonance of the gold surface. The effect of this heating on cell viability was also determined, the results from this study were in line with those from the ELISA measurement of heat shock proteins. At 50 µg·mL^−1^ HNP, cell viability fell to 85% after multiple laser irradiations. Upon intra-tumoral injection in pancreatic xenograft models, a similar heating capacity was observed at identical concentration which also resulted in bulk tumour dissipation [158].

**Figure 7:**
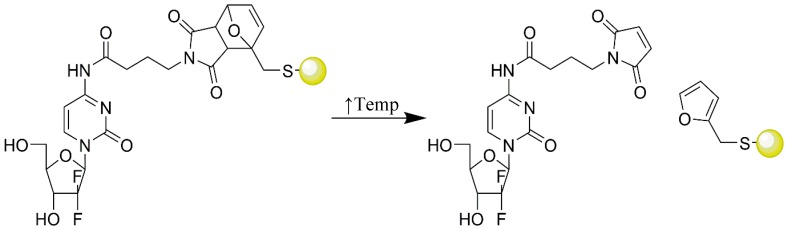
Schematic representation of hybrid nanoparticle with gemcitabine coupled onto its surface via a Diels Alder cycloadduct and its subsequent retro Diels Alder reaction after heating, liberating free gemcitabine.

Recently, Malekigorji et al. reported the use of hybrid nanoparticles as thermally triggered delivery systems for novel bisnaphthalimide based chemotherapeutics [56]. The novel drugs were attached onto the surface of the hybrid nanoparticles and the threshold temperature for release determined. The findings showed that, bisnaphtalimido propyl spermine tetrahydrobromide (BNIPSpm) was capable of binding onto the nanoparticle surface 3:1:0.25 drug:Fe:Au. At 44 °C, reversal of drug binding occurred. The novel formulation was tested on BxPC-3 cells in vitro and showed a 12-fold reduction in IC_50_ after 24 h. In vivo a 5-fold increase in tumour retardation was experienced after irradiation of the pancreatic xenografts in nude mice [56]. In another study, Oluwasanmi et al. developed a system whereby gemcitabine is coupled covalently onto a hybrid gold-iron oxide nanoparticle through a thermally labile linker, which undergoes a retro Diels Alder reaction, in order to improve drug efficacy and specificity (Figure 7) [57]. Drug binding onto the hybrid surface through linker attachment was achievable through dative covalent binding of a thiol in the linker onto the gold surface of the nanoparticle. Upon heating (via laser irradiation, 1064 nm, 20 s) it was shown that rapid drug release occurs which resulted in 62% reduction in tumour size when the formulation was introduced intra-tumorally into a pancreatic xenograft model in nude mice. The exciting data highlights the ability of the HNP-formulation to improve the cytotoxicity against PC adenocarcinoma compared with the free drug and also promote tumour retardation more efficiently [57].

One common problem particularly affecting the use of inorganic nanoparticles for cancer therapy is their higher relative toxicities [160] and accumulation within tissue [161] compared to small molecules or biodegradable polymers. Commonly, reports have shown that inorganic nanoparticles become trapped in the reticuloendothelial system and the liver [162]. This is usually due to proteins adhering onto their surface forming a corona which largely increases their hydrodynamic diameter [163]. In order to overcome this issue, studies have been carried out in order to coat these inorganic particles with various stealth materials such as poly(ethylene glycol) [164]. which not only can improve blood circulation times but also ability for renal clearance. Overcoming this issue is the key hurdle for clinical translation of many of the inorganic nano-therapies under development.

## 5. Conclusions and Future Perspectives

As highlighted in this review, the use of carefully formulated nanotechnologies as drug carriers or therapeutic agents has grown in traction for PC therapy. There is great promise in this field, a small number of nanotechnology formulations have made it through to the clinic for cancer therapy and it is expected that many more will follow within the next 10–15 years. Currently Abraxane^®^ is the only nanotechnology (1–300 nm) which has made it through clinical trial and onto the market for PC. Recently, in the UK, Abraxane^®^ was not available to everyone through the National Health Service, as the nanomedicine was deemed too expensive. This decision has since been overturned. However, this instance does need to be addressed when developing sophisticated new technologies. If nanotechnology is to have a huge impact on this disease and on the lives of its victims, the products hitting the market must be affordable. Indeed, there are also a lot of unknowns surrounding nanotechnologies in medicine, such as where do the formulations end up, how are they excreted, are they excreted, is there any long-term effects on accumulation or use in humans. Only further research and time will tell. Ultimately the goal for these studies is to improve patient lives, any improvement in PC therapy is significant as the outlook is so bleak. It is our belief that nanotechnology and nanomedicines provide the solution to many problems faced by current treatment strategies. These can be through earlier detection, increasing drug solubility, prolonging circulation, improving penetration into solid tumours, drug targeting controlling release duration and/or triggering release. The multifaceted approach to many of the systems under development are overcoming these physiological barriers step by step, with the hope that, in time, PC patients will face a brighter future upon diagnosis with improved quality of life and reduced recurrence of cancer.

## Figures and Tables

**Table 1:** Nanoparticle based drug delivery systems for PC therapy.

Type of Nano-System	Name of Nano-System	Drug Formulated	Testing Phase	
Polymer-drug conjugate	Poly(ethylene glycol)-P(HEMASN38)	SN38	Preclinical: In vivo	[39]
	Poly (TPGS)-PEG-GEM	Gemcitabine	Preclinical: In vitro	[40]
	Methacrylate-based GEM-monomer conjugate 3	Gemcitabine	Preclinical: In vitro	[41]
	Poly(ethylene glycol)-block-poly(2-methyl-2-carboxyl-propylenecarbonate)-graft-dodecanol-graft-cationic ligand	Gemcitabine	Preclinical: In vivo	[42]
Block copolymer	Styrene-maleic acid	CDF	Preclinical: In vitro	[43]
	Poly(ethylene glycol)-*b*-poly(glutamic acid)	Oxaliplatin	Preclinical: In vivo	[44]
Mixed micelles	Poly(vinyl pyrrolidone-b-polycaprolactone) (PVP-*b*-PCL) and poly(vinyl pyrrolidone-*b*-poly(dioxanone-co-methyl dioxanone)) (PVP-*b*-P(DX-co-MeDX)	Gemcitabine, doxorubicin, doxorubicin hydrochloride, 5-fluorouricil and paclitaxel	Preclinical: In vitro	[45]
Graft polymer	Poly(allylamine)-*g*-cholesterol	BNIPDAoct	Preclinical: In vivo	[46]
Dendrimer	PAMAM—hyaluronic acid	CDF	Preclinical: In vitro	[47]
	Poly(ethylene glycol)—PAMAM—poly(ethylene glycol)-Flt-2	Gemcitabine Hydrochloride	Preclinical: In vivo	[48]
Thermo-responsive polymer	Poly(diEGMAco-OEGMA300)-*b*-PEHMA	Squalenoyl-gemcitabine	Preclinical: In vitro	[49]
pH-responsive polymer	Poly(styrene-alt-maleic anhydride)	Curcumin	Preclinical: In vitro	[50]
Ultrasound-responsive nano-emulsion	PEG-PLLA	Paclitaxel	Preclinical: In vivo	[51]
Albumin	Abraxane^®^	Paclitaxel	FDA approved 2013	[52]
	Abraxane^®^/Gemcitabine	Paclitaxel & gemcitabine	Phase III	[53]
Inorganic nanoparticle	Iron oxide-dextran-DOX	Doxorubicin	Preclinical: In vitro	[54]
	Iron oxide-antiCD47-GEM	Gemcitabine	Preclinical: In vitro	[55]
	Iron oxide-gold	BNIPDSpm	Preclinical: In vivo	[56]
	Iron oxide-gold-GEM	Gemcitabine	Preclinical: In vivo	[57]
